# Diseases of the Throat

**Published:** 1872-08-15

**Authors:** 


Diseases of the Throat. A Guide to the Diagnosis and Treatment of Affections of the Pharynx, Æsophagus, Trachea, Larynx and Nares, by J. Solis Cohen, M. D. Wm. Wood & Co., Publishers, New York. For sale by Jansen, McClurg & Co., Chicago.

This is a handsome volume of five hundred and eighty pages, numerously illustrated with wood cuts.

The opening chapters are devoted to a general consideration of throat affections, methods of examination, the use of the laryngoscope, etc.

The different varieties of sore throat and the special affections of the Larynx and Trachea, etc., are then considered in detail under the following headings: Erythematous, Phlegmonous, Ulcerated and Membraneous Sore Throats, Diphtheria, The Sore Throats of the Exanthematous Affections, Syphilitic Sore Throat, Sore Throat from Burns and Scalds, Special Affections of the Palate and Uvula, Special Affections of the Pharynx, Special Affections of the Æsophagus, Affections of the Nasal Passages, Affections of the Frontal Sinus, Affections of the Larynx and Trachea, and a concluding chapter on Diseases of the Neck, affecting the deeper tissues of the throat secondarily.

The volume forms altogether the most complete and comprehensive treatise on this troublesome, and much neglected, class of affections that has ever been presented to the American profession. While many of the ideas and opinions advanced by the author are original with him, and derived from his own extensive experience in the treatment of these special affections, the major part of the volume is made up of compilations from all the more modern treatises on the subject.

